# Is Extracellular Vesicle-Based Therapy the Next Answer for Cartilage Regeneration?

**DOI:** 10.3389/fbioe.2021.645039

**Published:** 2021-04-23

**Authors:** Émilie Velot, Henning Madry, Jagadeesh K. Venkatesan, Arnaud Bianchi, Magali Cucchiarini

**Affiliations:** ^1^Faculté de Médecine, Biopôle de l’Université de Lorraine, Campus Brabois-Santé, Laboratoire UMR 7365 CNRS-Université de Lorraine, Ingénierie Moléculaire et Physiopathologie Articulaire (IMoPA), Université de Lorraine, Vandoeuvre-Lès-Nancy, France; ^2^Campus Brabois-Santé, Laboratoire de Travaux Pratiques de Physiologie, Faculté de Pharmacie, Université de Lorraine, Vandoeuvre-Lès-Nancy, France; ^3^Center of Experimental Orthopaedics, Saarland University, Homburg, Germany

**Keywords:** extracellular vesicles, cell-to-cell communication, stem cells, regenerative medicine, cartilage regeneration, cell-free therapy

## Abstract

“Extracellular vesicles” (EVs) is a term gathering biological particles released from cells that act as messengers for cell-to-cell communication. Like cells, EVs have a membrane with a lipid bilayer, but unlike these latter, they have no nucleus and consequently cannot replicate. Several EV subtypes (e.g., exosomes, microvesicles) are described in the literature. However, the remaining lack of consensus on their specific markers prevents sometimes the full knowledge of their biogenesis pathway, causing the authors to focus on their biological effects and not their origins. EV signals depend on their cargo, which can be naturally sourced or altered (e.g., cell engineering). The ability for regeneration of adult articular cartilage is limited because this avascular tissue is partly made of chondrocytes with a poor proliferation rate and migration capacity. Mesenchymal stem cells (MSCs) had been extensively used in numerous *in vitro* and preclinical animal models for cartilage regeneration, and it has been demonstrated that their therapeutic effects are due to paracrine mechanisms involving EVs. Hence, using MSC-derived EVs as cell-free therapy tools has become a new therapeutic approach to improve regenerative medicine. EV-based therapy seems to show similar cartilage regenerative potential compared with stem cell transplantation without the associated hindrances (e.g., chromosomal aberrations, immunogenicity). The aim of this short review is to take stock of occurring EV-based treatments for cartilage regeneration according to their healing effects. The article focuses on cartilage regeneration through various sources used to isolate EVs (mature or stem cells among others) and beneficial effects depending on cargos produced from natural or tuned EVs.

## Introduction

With an aging population, musculoskeletal diseases remain a worldwide challenge, both economical and therapeutic, for public health (Woolf and Pfleger, 2003; Sanchez-Adams et al., 2014; Hunter and Bierma-Zeinstra, 2019). Among these diseases, those including articular cartilage degeneration are very deleterious by contributing to the societal burden through disability and morbidity (Woolf and Pfleger, 2003; Hunter and Bierma-Zeinstra, 2019; Komarraju et al., 2020). Cicuttini et al. (2005) examined a population of healthy, middle-aged subjects. The authors demonstrated that individuals with cartilage defects are affected by a loss of articular cartilage compared with their initial cartilage volume. This loss of cartilage represents a 2.5% annual rate of loss in terms of cartilage volume. However, a pathological early degeneration of articular cartilage can ensue following osteoarthritis (OA), trauma, or other causes (infections, hereditary conditions, etc.) (Komarraju et al., 2020). OA is estimated to be the most common affliction triggering cartilage degeneration by affecting more than 300 million patients all around the world and more than 40% of the elderly older than 70 years (Hunter and Bierma-Zeinstra, 2019; Magnusson et al., 2019; Kolasinski et al., 2020). The mechanisms causing OA are still not fully understood, and the global therapeutic strategy is to maintain the patient quality of life mainly by treating symptoms (i.e., the consequences of cartilage degeneration) (Hunter and Bierma-Zeinstra, 2019; Hsu and Siwiec, 2020; Kolasinski et al., 2020; Oliveira Silva et al., 2020).

Articular cartilage is a hyaline cartilage covering the surface of bones in diarthrodial joints. This specialized connective tissue is aneuronal and avascular, meaning it does not have its own blood supply. Therefore, its nutrition depends on synovium through the imbibition from synovial fluid or subchondral bone, which blood supply is brought to cartilage bounds (Oliveira Silva et al., 2020). The main cell type constituting cartilage is called chondrocyte. Chondrocytes are surrounded by an extracellular matrix (ECM) they synthetize. Articular cartilage viscoelastic properties provide wear-resistant surfaces to the joint by improving the distribution of mechanical loads and moderating the friction between its surfaces (Sanchez-Adams et al., 2014; Lindahl, 2015; Bianchi et al., 2020; Masson and Krawetz, 2020). When injured or degenerating, articular cartilage is not able to restore its original organization because it has a very weak regenerative capacity. The lost hyaline cartilage is replaced by fibrocartilage, but this reparative process is mechanically inadequate with a new cartilage lacking inherent functionality (Goldring et al., 2017; Lepage et al., 2019; Masson and Krawetz, 2020).

OA is a disease related to cartilage degradation, osteophyte formation, and subchondral bone alteration. It leads gradually to a joint destruction, which generates severe impairment of mobility for the patient (Lindahl, 2015; Hsu and Siwiec, 2020). The management of OA patients mostly starts with non-surgical treatments that are replaced by surgical ones if losing their efficacy. Usually, pain management is prioritized over other treatments for early stages OA, whereas invasive surgery is preferred for late stages (Hsu and Siwiec, 2020; Yu and Hunter, 2020). There is still no pharmacological treatment that would be able to repair or regenerate cartilage (Chevalier et al., 2013). Cartilage repair can be enhanced by surgery as described hereinafter. Conventional surgical approaches include methods such as subchondral bone microfracture, soft tissue transplantation with an autograft of periosteum/perichondrium, osteochondral allograft/autograft transplantation, and autologous chondrocyte implantation (Bakhshayesh et al., 2020). When effective, these methods often lead to the production of fibrocartilage instead of hyaline cartilage. It is important to integrate that repair is different from regeneration. Repair means healing joint through fibrocartilage synthesis, resulting in a tissue with poorer functional quality that is not mechanically equivalent to native hyaline cartilage and cannot restore full original function. Besides, end-stage OA leads to invasive surgery with total joint replacement by limited life prostheses (Chard et al., 2005; Lindahl, 2015; Kolasinski et al., 2020).

Regenerative medicine strategies have been initiated as a new opportunity to treat cartilage defects and bring regeneration along. Numerous tissue engineering approaches have been developed by using biomaterials as ECM derivatives or mesenchymal stem cells (MSCs) as chondrogenic precursors to alleviate the lack of accessibility for primary chondrocytes (Bakhshayesh et al., 2020; Tsiapalis and O’Driscoll, 2020). Because of their promising results *in vitro* or on animal models, these approaches are currently investigated to be transferred to clinic practice (Borić et al., 2019; Kim et al., 2020; Kyriakidis et al., 2020; Lehoczky et al., 2020; Mendes et al., 2020). Cell therapy approaches involving patients are also encouraging, and many clinical trials are ongoing. Larger sample sizes and long-term follow-ups would be necessary for a validation in the clinics. In addition, the proof of effectiveness for intra-articular cell injection remains limited in terms of repair as much as regeneration (Ha et al., 2019; Monckeberg et al., 2019; Lee et al., 2019; Zhao et al., 2019).

Other regenerative medicine strategies involved cell-free therapies by delivering exogenous active biomolecules (growth factors, drugs, etc.) as therapeutic agents directly through intra-articular injection. The flaws of this route are that most mediators have a short half-life and are promptly removed from the joint space and that mediators cannot reach chondrocytes suitably because these cells are embedded into a thick avascular ECM (Yan et al., 2019; Eckstein et al., 2020). The first flaw can be moderated by encapsulating the mediator in an appropriate scaffold to ensure a gradual diffusion inside the joint and a more long-lasting effect, but the ECM penetration to get to chondrocytes still remains challenging (Szychlinska et al., 2018).

The cells, among which MSCs are the most solicited, used in tissue engineering or cell therapy are supposed to have intrinsic capabilities to mediate tissue repair. For example, numerous studies have now demonstrated that MSC healing effect is a paracrine effect due to cell secretome (Julianto and Rindastuti, 2016; Khatab et al., 2018; Chen et al., 2019b; Niada et al., 2019; Mancuso et al., 2019; Brennan et al., 2020; Parate et al., 2020; Ragni et al., 2020b; Kadir et al., 2021). The secretome includes all cell secretions, i.e., soluble mediators (growth factors, cytokines, etc.) (Maumus et al., 2017) and also very unique entities called extracellular vesicles (EVs) (Dostert et al., 2017; Tsiapalis and O’Driscoll, 2020).

Since the first decade of the twenty-first century, literature about EVs has been growing exponentially. But in parallel, a lack of accuracy for certain publications was noticed by experts in the field. A new scientific community in need of dogma developed around EV research leaders resulting in the foundation of the International Society for Extracellular Vesicles (ISEV) in 2012 (Witwer and Théry, 2019). EVs are commonly described as membranous entities released by every cell type into extracellular space. They are not able to replicate and are involved in cell-to-cell communication. Most publications depict EVs as particles enriched with components from the releasing cell and bordered by a lipid bilayer comparable to the plasma membrane (Yáñez-Mó et al., 2015). Many subtypes of cell-released structures are encompassed in the term “EV” [e.g., small/medium/large vesicles, exosomes, ectosomes, oncosomes, microvesicles (MVs), microparticles, apoptotic bodies (ABs), matrix vesicles, etc.]. Consequently, the whole EVs constitute hard-to-characterize heterogeneous populations. The worldwide harmonization on “how to term” the various EV subtypes has still not been set, and their designation could depend on the size, biogenesis, specific markers, location or origin (ECM, cell/tissue, tumor, etc.), and/or conditioning (identified culture conditions). This lack of consensus can sometimes make the designation of EV subtypes ambiguous (Théry et al., 2018; Witwer and Théry, 2019).

Three categories of EVs are frequently found in publications and are described according to biogenesis. ABs are large EVs (±1–5 μm) released after dismantling of apoptotic cells and made of subcellular fragments. MVs, also termed ectosomes or microparticles, are medium/large EVs (±100 nm to 1 μm) materialized from the budding of the plasma membrane. Exosomes are small EVs (±30–150 nm depending on the authors) derived from endocytic pathway (Dostert et al., 2017; Witwer and Théry, 2019; Cocozza et al., 2020; Tsiapalis and O’Driscoll, 2020).

In 2014, ISEV set up guidelines called minimal information for studies of EVs (MISEV), which were updated in 2018. MISEV were a necessary step to support scientists interested in this complex and still evolving field and to clarify/prevent the confusion about EV designation. MISEV2018 define “EV” as a generic term. The authors are free to choose the required approaches to characterize EV subtypes. However, if they cannot match the guidelines to validate EV identity, they are requested to use the term extracellular particle or to find their own term after setting a clear definition (Théry et al., 2018). For example, extracellular mitochondria alone or encapsulated in vesicles constitute a noticeable subset of EVs (Puhm et al., 2019; Al Amir Dache et al., 2020). Likewise, Yefimova et al. (2020), have recently designated a new kind of EVs found in seminal plasma called myelinosome. Myelinosomes are secretory organelles entirely released by Sertoli cells through a process close to endocytic pathway. These newly discovered EVs have a multilamellar demarcation very different from the lipid bilayer typically encountered in EVs (Yefimova et al., 2020).

EVs have many interesting assets that could improve cartilage defects. They have the innate capacity to target difficult-to-reach places by crossing the blood–brain barrier or ECM. Their membranous structure allows protecting their cargo from the environment. According to their sources, they have immunomodulatory properties and are biocompatible (Tsiapalis and O’Driscoll, 2020). All these benefits offer new insights to use EVs as regenerative medicine tools to heal cartilage.

This mini review summarizes current EV-based treatments to improve cartilage degeneration by focusing on various sources to produce EVs and the positive effects of the therapeutic cargos from natural or modified EVs.

## Biological Source-Derived EVs for Cartilage Healing

EVs participate in the modulation of numerous cell regulatory processes (e.g., proliferation, differentiation, or inflammation), making these membranous entities perfect stakeholders for tissue regeneration according to their various sources (Dostert et al., 2017; Tsiapalis and O’Driscoll, 2020). EVs are secreted by diverse cell types and found in solid tissues (e.g., ECM) or in body fluids (blood, saliva, urine, milk, etc.). They can be isolated from these biological samples, but determining their exact cell origin is not always possible because of their heterogeneity. According to the use of EVs, particularly for therapeutic purpose, their availability in sufficient quantity must be demonstrated to prevent precious sample scarcity and to ensure the results obtained from their exploitation. Despite the restrictions linked to good manufacturing/clinical practice (GMP/GCP), cell culture is also a reasonable answer to overcome the lack of biological sources and to standardize an *in vitro* EV production from a well-defined cell type. It allows EV isolation from cell-conditioned medium (Théry et al., 2006, 2018; Grant et al., 2011; Chen et al., 2013; Wolf et al., 2015; Iwai et al., 2016; Palamà et al., 2020; Ragni et al., 2020b).

**FIGURE 1:**
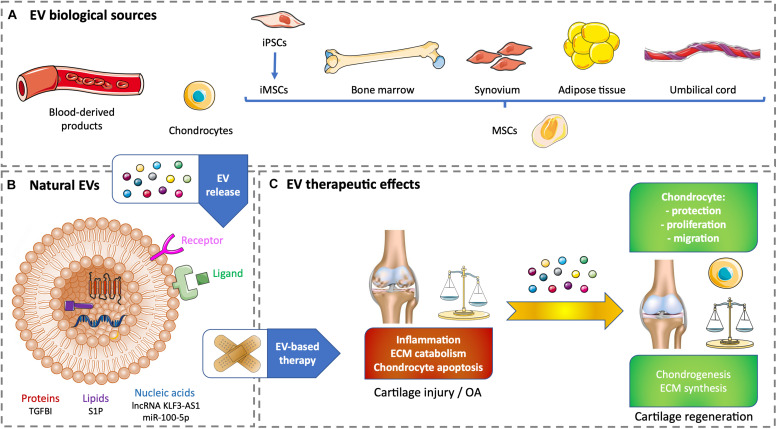
Therapeutic effects of natural EV-based therapy for cartilage healing. **(A)** Various cell sources for generating non-modified EVs. **(B)** Example of therapeutic cargos for natural EVs conveying proteins (TGFBI), nucleic acids (lnc KLF3-AS1, miR-100-5p), or lipids (SP1). **(C)** EV therapeutic effects on cartilage injury or OA. ECM, extracellular matrix; EV, extracellular vesicle; iPSC, induced pluripotent stem cell; iMSC, induced pluripotent stem cell-derived mesenchymal stem cell; lncRNA, long non-coding RNA; miR, microRNA; MSC, mesenchymal stem cell; OA, osteoarthritis; S1P, sphingosine-1-phosphate; TGFBI, transforming growth factor β–induced protein.

Blood products, such as serum- or plasma-based whole-blood derivatives, have a good tolerance and can reduce pain and inflammation. Intra-articular injection of blood products over other intra-articular treatments has been investigated and improves pain scales in knee OA. The benefits become statistically and clinically significant starting from 6 months and increase up to 12-month follow-up (Filardo et al., 2020). Whole blood non-invasively drawn from patients allows a direct conditioning to obtain autologous blood-derived products with a higher tolerance (Gato-Calvo et al., 2019). Among cell-free strategies to regenerate cartilage, EVs from autologous blood products have a stronger effect in OA chondrocytes than full blood products by influencing cartilage ECM metabolism and inflammation. Acute OA could be improved by the positive anti-inflammatory effect of citrate-anticoagulated platelet-rich plasma EVs. Chronic OA could benefit the chondrogenesis elicited by hyperacute serum EVs. These promising *in vitro* results have to be tested *in vivo* to demonstrate cartilage regeneration (Otahal et al., 2020; Figures 1A,C).

Current regenerative strategies for cartilage regeneration rely on the use of MSCs. These cells are multipotent with a chondrogenic potential. They also have self-renewal and immunomodulatory properties (Dostert et al., 2017; Tsiapalis and O’Driscoll, 2020). However, using growth factors to differentiate MSCs toward an articular phenotype remains difficult and can lead to hypertrophy associated with unwanted ossification (Deng et al., 2019). Lately, primary articular chondrocytes harvested from patients who underwent polydactyly surgery were used to produce EVs. The aim was to use chondrocyte-derived EVs (C-EVs) instead of growth factors to mediate MSC differentiation into chondrocytes. Human umbilical cord–derived MSCs (UCMSCs) were treated with C-EVs. *In vitro*, C-EVs promoted chondrogenic differentiation of MSCs and decreased fibrotic and hypertrophic markers. *In vivo*, MSCs conditioned by C-EVs were successfully used to repair knee cartilage defect in a rabbit model. This approach needs further developments to be tested for human therapeutic purpose. Its main flaws reside in the accessibility of mature chondrocytes and their tolerance in an allogeneic context (Ma et al., 2020; Figures 1A,C).

MSCs have a unique capacity to build a regenerative microenvironment, which is considered to promote chondrogenic differentiation in the case of cartilage injury through soluble mediators and EVs. *In vitro* and *in vivo* studies have demonstrated the immunomodulatory, chondroprotective, and regenerative effects of MSC-derived EVs (MSC-EVs), which is equivalent or superior to MSCs alone. Harvesting human adult MSCs (e.g., bone marrow, adipose tissue, or dental pulp) often requires invasive procedures, and these cells are not systematically available, particularly in sufficient amount for cell therapy or tissue engineering. Consequently, adult MSCs need to be expanded *in vitro*, which can lead to chromosomal aberrations. EV-based therapy is an answer to alleviate the previous hindrances and potential cell immunogenicity. The stemness of adult MSCs decreases with the increase of the donor age. Adult MSCs have limited self-renewal and immunomodulation properties compared with perinatal MSCs (e.g., cord blood or umbilical cord connective tissue, also known as Wharton jelly) or MSCs derived from embryonic stem cells. Perinatal MSCs are easily harvested, whereas embryonic MSCs can cause ethical issues. Induced pluripotent stem cells (iPSCs) have limitless self-renewal and can differentiate into MSCs to generate iPSC-derived MSCs (iMSCs). Nevertheless, the use of iPSCs or iMSCs in clinics is questionable because of their possible genomic instability, immunogenicity, and tumorigenicity (Zhang et al., 2016; Dostert et al., 2017; Zhu et al., 2017; Tsiapalis and O’Driscoll, 2020). Whether it is from an allogeneic or an autologous origin, the best source of MSC-EVs has not been yet highlighted. Several examples of MSC-derived EV therapy will be illustrated in the following sections of this article. To date, there is only one clinical trial involving EVs to treat cartilage injury. It is based on the promising results from Niada et al. (2019), where MSCs derived from adipose tissue of healthy donors undergoing aesthetic or prosthetic surgery. This observational study has been posted in January 2020, and there is still no recruitment (National Clinical Trial no. NCT04223622)^1^. One of its purposes is to validate a cell-free approach based on the use of EVs produced by adipose tissue–derived MSCs (ATMSCs) to improve an *ex vivo* OA model. Besides, one of the possible sources of autologous ATMSCs could come from OA patients’ infrapatellar fat pad (IFP) obtained after arthroscopy, although such cells may be compromised in their regenerative potential as the IFP has been involved in the pathogenesis of OA. Autologous MSCs from OA patients’ joint tissues such as the synovium or IFP could originate from an inflammatory environment characterized by progressive OA causing their potential priming by proinflammatory factors. This priming does not seem to compromise the healing aptitudes of MSCs. Instead, Kouroupis et al. (2019) showed that primed MSCs derived from the IFP exhibit enhanced immunomodulatory properties *in vitro* and *in vivo* (Figures 1A,C).

## Therapeutic Effects of Regular EVs for Cartilage Healing

The therapeutic effects of EVs depend on the cargo they conveyed. Indeed, tissue regeneration relies on healing factors carried by EVs to generate a trophic microenvironment suitable for chondrogenesis and hyaline ECM upkeep (Liu et al., 2017; Tao et al., 2017; Jing et al., 2020; Zhao et al., 2020). EVs can be untouched and used as therapeutic agents in their regular or natural state after isolation from biological samples or conditioned medium in standard cell culture conditions (Figure 1).

The following studies highlight the molecules of interest conveyed by therapeutic EVs that could become part of the future strategies used for cartilage healing. Transforming growth factor β (TGF-β) is a major regulator of cartilage homeostasis, and the deregulation of its pathway is involved in OA. A member of the TGF-β family, the TGF-β–induced protein (TGF-BI), is upregulated in bone and cartilage of OA patients, but is downregulated in human bone marrow–derived MSCs (BMMSCs) (Ruiz et al., 2019). Recently, Ruiz et al. (2020), showed that TGBI silencing inhibits murine BMMSCs’ chondroinductive effect *in vitro* and healing effect in a collagenase-induced OA mouse model. These positive effects are due to the presence of TGF-BI mRNA and protein in BMMSC-derived EVs, suggesting that TGF-BI is a new key factor released by MSCs to protect cartilage and favor its anabolism (Ruiz et al., 2020). The long non-coding RNA (lncRNA) KLF3-AS1 (see Ref seq NR_026804.1) is found in human MSCs and their EVs. When used in an *in vitro* OA model based on rat articular chondrocytes treated with the proinflammatory cytokine interleukin 1β (IL-1β), EVs carrying lncRNA KLF3-AS1 suppress IL-1β–induced apoptosis of chondrocytes. Additionally, these EVs engender cartilage repair and chondrocyte proliferation in a knee collagenase–induced OA rat model (Liu et al., 2018b). EV subtypes secreted by human BMMSCs contain a high level of sphingosine-1-phosphate (S1P) compared with MSCs alone. This enrichment is due to the cell enzyme sphingosine kinase 1. S1P-enriched EVs enhance the proliferation of human chondrocytes and inhibit IL-1β–induced apoptosis *in vitro*. In a rabbit model of knee articular cartilage injury made by drilling, the injection of these EVs into the knee capsule promotes the recovery of the cartilage defect (Xiang et al., 2018). OA patients’ ATMSCs derived from IFP released EVs conveying high levels of miR-100-5p. These EVs inhibit cell apoptosis, promote ECM anabolism, and repress ECM catabolism in an *in vitro* OA model made of OA patients’ chondrocytes treated with IL-1β. Intra-articular injections of these ATMSC-EVs in a mouse model based on knee joint instability induced by surgery enhance articular cartilage protection from damage and improve gait abnormality due to OA pain and disturbance (Wu et al., 2019; Figures 1B,C).

One of the limitations to have information on EV cargos is that numerous studies characterize EV effects and not their content or membrane composition. Moreover, the differences between cell type and cell donor have to be taken into account to ensure the potential of the therapeutic message conveyed by EVs (Ragni et al., 2019). The following examples illustrate the positive use of natural EVs on cartilage without deciphering their cargos. It was previously mentioned that blood product–derived EVs beneficially influence cartilage ECM metabolism and inflammation *in vitro* (Otahal et al., 2020) and that articular C-EVs favor chondrogenic differentiation of human UCMSCs *in vitro* and *in vivo* (Ma et al., 2020). Besides, human embryonic MSC-derived EVs promote cartilage regeneration in an adult rat cartilage defect model (Zhang et al., 2016). In an OA model based on rabbit chondrocytes treated with IL-1β, EVs secreted by rabbit BMMSCs prevent chondrocyte mitochondrial-induced apoptosis in response to IL-1β *in vitro* (Qi et al., 2019). Human BMMSCs produce chondroprotective and anti-inflammatory EVs *in vitro* that prevent mice to develop collagenase-induced OA (Cosenza et al., 2017; Vonk et al., 2018). EVs secreted by human synovial membrane–derived MSCs (SMMSCs) and human iMSCs are able to attenuate OA in the same animal model. However, human iMSC-EVs show a superior therapeutic effect associated with an improvement of chondrocyte migration and proliferation (Zhu et al., 2017). Intra-articular injections of human iMSCs-derived EVs also allow ECM restoration and cartilage regeneration in a rat temporomandibular joint OA model induced by monosodium iodoacetate (Zhang et al., 2019). In addition, EVs secreted by human iMSCs alleviate OA in the mouse, *in vitro* in chondrocytes treated with IL-1β and *in vivo* by limiting cartilage destruction and matrix degradation according to Osteoarthritis Research Society International (OARSI) scores in a model based on knee joint instability induced by surgery (Wang et al., 2017). Human ATMSCs derived from abdominoplasty release EVs that decrease the inflammation caused *in vitro* by IL-1β in patient-derived OA chondrocytes (Tofiño-Vian et al., 2018; Figures 1A,C).

Furthermore, EVs can also be tuned to carry selected mediators favoring a specific enrichment to elicit cartilage regeneration. On the one end, engineered or primed tissues/cells can lead to the production of enriched EVs. On the other hand, EVs can also be loaded after their isolation.

## Therapeutic Effects of Tuned EVs for Cartilage Healing

**FIGURE 2:**
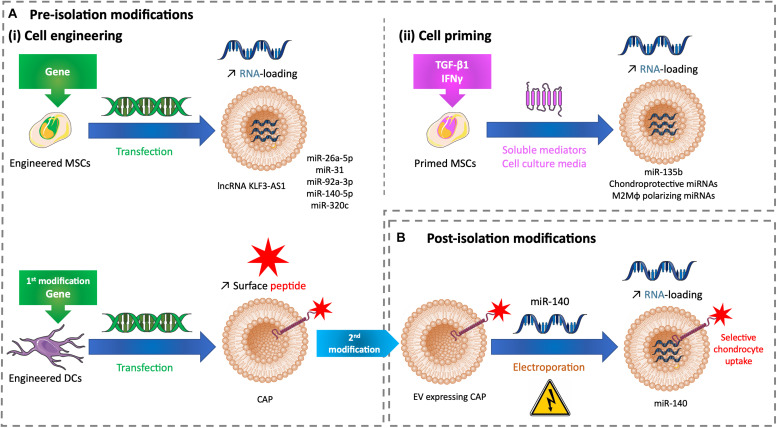
Tuning methods to improve the drug loading of therapeutic EVs for cartilage healing. **(A)** Methods of modifications used before EV isolation to tune EVs with **(i)** cell engineering by transfecting cells or **(ii)** cell priming by treating cells with soluble mediators added to cell culture medium. **(B)** Methods of modifications used after EV isolation to tune EVs with miRNA electroporation. DC, dendritic cell; EV, extracellular vesicle; CAP, chondrocyte affinity peptide; IFN-γ, interferon γ; lncRNA, long non-coding RNA; M2Mφ, M2 macrophage; miR, microRNA; miRNA, microRNA; MSC, mesenchymal stem cell; TGF-β1, transforming growth factor β1.

Tuning EVs to increase their therapeutic potential require EV drug-loading strategies. Priming or transfecting cells involved an EV loading performed by the donor cell, which secrete EVs. Postisolation drug loading occurs after EV secretion by the donor cell. EV-based therapy for cartilage regeneration with tuned EVs depends mainly on RNA loading from transfected MSCs (Figure 2).

As previously described, lncRNA KLF3-AS1 conveyed by MSC-EVs improves OA *in vitro* and *in vivo* by respectively, suppressing IL-1β–induced apoptosis of chondrocytes and eliciting cartilage repair and chondrocyte proliferation in a knee collagenase-induced OA model (Liu et al., 2018b). The role of lncRNAs is to act as competitive endogenous RNA (ceRNAs) to separate microRNAs (miRNAs) from target mRNAs, and thus restoring mRNA expression. Among miRNAs, miR-206 is known to be highly expressed in OA. When transfected to overexpress lncRNA KLF3-AS1, MSCs release EVs with a high load of this lncRNA. These enriched EVs used in similar *in vitro* and *in vivo* models showed a significant improve of IL-1β–induced chondrocyte apoptosis and cartilage injury. The authors demonstrated that lncRNA KLF3-AS1 conveyed by MSC-EVs acts as a ceRNA to prevent miR-206 to downregulate G-protein-coupled receptor kinase interacting protein-1 (GIT1) expression, which induces EV therapeutic effects. Indeed, GIT1 inhibits chondrocytes apoptosis and promotes chondrocytes proliferation. Delivering this lncRNA by EVs into cartilage could be a future strategy for cartilage regeneration (Liu et al., 2018a; Figure 2Ai upper panel).

In an OA rat model based on knee joint instability induced by surgery, human SMMSCs promote to a lesser extent the same positive effects -as described previously- but inhibit the synthesis of chondrocyte ECM (Tao et al., 2017). The microRNA (miR)-140-5p is known to play a role in chondrogenic differentiation and OA prevention (Tardif et al., 2009; Barter et al., 2015). SMMSC-derived EVs contain this miRNA weakly. When modified to overexpress miR-140-5p, SMMSCs secrete EVs enriched in miR-140-5p. This packaging enhanced the healing properties of human SMMSC-EVs by delaying the progression of early stage OA and cartilage damage (Tao et al., 2017). Likewise, miR-26a-5p and miR-31 promote chondrocyte proliferation and improve OA. Respectively, human BMMSCs and human SMMSCs overexpressing miR-26a-5p and miR-31 secrete EVs enriched in these miRNAs. These EVs alleviate cartilage destruction, matrix degradation, and inflammation in OA models based on knee joint instability induced by surgery, in the rat for EVs carrying miR-26a-5p according to the authors’ subjective scores based on synovitis inflammation, synovial thickening, and subchondral bone erosion (Jin et al., 2020) and in the mouse for EVs carrying miR-31 according to OARSI scores (Wang et al., 2020). EVs derived from rat BMMSCs transfected to overexpress miR-135b favor rat chondrocyte proliferation *in vitro* (Wang et al., 2018). Human BMMSCs overexpressing miR-92a-3p, a chondrogenesis promoter and cartilage degradation inhibitor (Mao et al., 2017), produce EVs sustaining its properties *in vitro*. Human BMMSC-EVs enriched in miR-92a-3p interfere with the progression of early-stage OA and prevent cartilage damage in a knee collagenase–induced OA mouse model (Mao et al., 2018). Human BMMSCs undergoing chondrogenic differentiation upregulate the EV loading of specific miRNAs, like miR-320c. EVs derived from human BMMSCs transfected to overexpress miR-320c promote OA patients’ chondrocyte proliferation and migration and increase human BMSC chondrogenesis (Sun et al., 2019). The previous studies demonstrate the potential of miRNA-loaded EVs to develop novel therapeutic strategies for cartilage healing (Figure 2Ai upper panel).

Palamà et al., showed that human BMMSCs grown in a xeno-free culture system produce chondroprotective EVs *in vitro*. In addition, these EVs were produced in a higher amount and inhibit the adverse effects of IL-1α-induced inflammation in an *in vitro* OA model compared with cells grown in a conventional culture system with fetal bovine serum (Palamà et al., 2020). This demonstrates that a xeno-free environment can prime cells and influence EV composition and consequently EV effects. Similarly, cell priming with biochemical factors influences EV cargos. EVs derived from rat BMMSCs primed with TGF-β1 are enriched in miR-135b and favor rat chondrocyte proliferation by upregulating S1P *in vitro* (Wang et al., 2018). Moreover, these EVs engender cartilage repair in a rat OA model based on knee joint instability induced by surgery. Interferon γ (IFN-γ) is a versatile proinflammatory cytokine involved in tissue regeneration. *In vitro* IFN-γ–primed human ATMSCs secrete EVs enriched in chondroprotective and M2 (i.e., anti-inflammatory) macrophage polarizing miRNAs (Ragni et al., 2020b). This cargo improvement makes a suitable association to promote cartilage regeneration (Figure 2Aii).

The delivery of EVs after intra-articular administration in chondrocytes remains challenging as these cells are surrounded by an avascular, dense ECM. The density of hyaline ECM is due to proteoglycans with high negative charges entangled in a collagen fibril network. Only solutes less than or equal to 10 nm have been shown to cross this thick barrier and penetrate the cartilage (Bajpayee et al., 2014). Although EVs are greater than 10 nm and have a slight negative charge that could push them away from cartilage, the previous references showed their healing effects on damaged cartilage whether they are regular or tuned. However, their penetration in the hyaline ECM has not always been tested. Recently, Liang et al. (2020), transfected dendritic cells to produce engineered EVs that are tagged with a chondrocyte affinity peptide (CAP) at their surface. CAP-EVs have the same size range (40–200 nm) as EVs without CAP. CAP-EVs target and enter selectively chondrocytes *in vitro* and *in vivo*. They can more efficiently and deeply penetrate healthy rat cartilage than non-tagged EVs that mainly reside in the surface layer. After their isolation, CAP-EVs and non-tagged EVs were loaded with miR-140 mimic by electroporation. Loaded CAP-EVs were able to improve an *in vitro* OA model based on human articular chondrocytes treated with IL-1β by a targeted delivery of miR-140. Intra-articular injection of EVs was made in an OA rat model based on knee joint instability induced by surgery. CAP-EVs remained preferentially into cartilage with minimal diffusion compared with non-tagged EVs. Loaded CAP-EVs delivered miR-140 to deep cartilage areas and prevent cartilage degradation and OA progression (Liang et al., 2020; Figure 2Ai lower panel, Figure 2B).

EVs can penetrate deep down into tissue, and their faintly negative net charge allows long circulation in the body. Moreover, they are able to escape immune cells by avoiding clearance or degradation (Malhotra et al., 2016; Vader et al., 2016). These assets made them ideal biocompatible carriers to convey molecules of interest for cartilage healing. Whatever EV enrichment, all of these molecules have not yet been discovered, and there is still an in-depth screening ongoing to bring them up to light by the scientific community (Endisha et al., 2018; Sun et al., 2019; Ragni et al., 2020a).

## Discussion

Although a lack of consensus concerning EV isolation methods and EV characterization/designation remains, EV therapeutic potential cannot be denied to improve cartilage injury as shown in the preclinical studies presented in this article. Cell-free therapy based on EVs appears to demonstrate similar or sometimes even better regenerative properties of cartilage compared with cell therapy. For example, phosphate-buffered saline (PBS), EVs derived from human BMMSCs, or human BMMSCs were injected in the knee capsule in a rabbit model of knee articular cartilage injury performed by drilling. The control group treated with PBS exhibited defect filling with adipose and fibrotic cells but without ECM, suggesting a poor cartilage repair. The EV-treated group or the MSC-treated group displayed contrasting results, with defects progressively filled by a hyaline cartilage–like tissue. There was no significant difference between the EV-treated and MSC-treated groups, indicating that EVs are as efficient as MSCs to promote the recovery of cartilage defects (Xiang et al., 2018). Similarly, intra-articular injection of human BMMSC full secretome (containing EVs), equally to injection of human BMMSCs, was shown to reduce pain and have protective effects on the development of cartilage damage in a knee collagenase–induced OA mouse model (Khatab et al., 2018). Cosenza et al. (2017) reported the superiority of human BMMSC-EVs over human BMMSCs to protect the joint from OA in the same *in vivo* preclinical model. Moreover, EVs are easier to handle and display minor regulatory concerns compared with cells, mainly because they are less immunogenic, and because they have no nucleus, they do not replicate. To translate EV-based therapy into clinics, several hurdles would have to be overcome to guarantee the procurement of safe therapy products and prevent side effects, such as EV cell sources/donor related to potential immunogenicity and EV dose and route related to pharmacokinetics, pharmacodynamics, and toxicity (Théry et al., 2018; Tieu et al., 2020).

Alongside the GMP/GCP, the future of patients’ cartilage regeneration appears to have a great potential within cell-free therapies based on EVs. EV aptitude to cross ECM allows them to reach and heal cartilage. However, to penetrate the cartilage, EVs seemed to need a special tag allowing to cross the hyaline ECM. While tuned EVs can be enhanced to carry an established tag (Liang et al., 2020), regular EV healing cartilage could be enriched and carry inherently those kinds of tags such as S1P (Xiang et al., 2018). The selective mechanisms to favor EVs and ECM interaction could rely on a chemotactic response (Headland et al., 2015). According to the disease state of OA and to the depth of the defects, EVs could also have an easier access to exposed chondrocytes without being hindered by the hyaline ECM, which is removed from cartilage by erosion.

EVs are used in their natural state or tuned to be drug-loaded in order to be enriched with cartilage regenerative RNAs (e.g., miRNAs or lncRNAs). The tuning can also apply to EV membrane to improve chondrocyte targeting (e.g., surface modification with CAP). To date, these modifications are the only existing for cartilage, despite a broad range of possibilities that would probably inspire forthcoming research (Liao et al., 2019). Strategies using cotherapy by tuning EVs with drug encapsulation can also be considered to boost cartilage regeneration. For example, loading EVs with pharmacological agents such as glucosamine, chondroitin sulfate, or non-steroidal anti-inflammatory drugs could improve ECM degradation and reduce pain and cartilage degeneration (Artuzi et al., 2020; Sun et al., 2020). Although liposomes can be used as synthetic vesicles to convey drugs to cells, it has been shown that they could be silenced through phagocytosis by monocytes and macrophages. Consequently, they require surface adjustments to obtain smart targeting abilities. EVs carry specific membrane proteins that protect them from phagocytosis and facilitate their delivery (Kamerkar et al., 2017). A strategy for cotherapy could also be to engineer “smarter” delivery systems by creating hybrid EV-liposome carriers with membrane fusion (Elkhoury et al., 2020).

Apart from cell-free therapy, EVs can also be used for cartilage tissue engineering strategies by being associated with implantable engineered constructs (Malda et al., 2016; Szychlinska et al., 2018). After being implanted subcutaneously in nude mice, constructs made of rabbit cartilage progenitor cells and alginate have developed into ectopic cartilage once injected with rabbit C-EVs (Chen et al., 2018). An acellular tissue patch made of hydrogel glue and human iMSC-EVs was tested in a rabbit model of patellar groove defect. The hydrogel glue could retain and then release EVs sustainably. The patch was easily integrated to cartilage ECM and improved the articular cartilage injury through cell deposition (Liu et al., 2017). The same animal model was used to test a three-dimensional (3D) construct printed with a bioink made of cartilage ECM, gelatin methacrylate, and BMMSC-EVs. The 3D construct stimulated chondrocyte migration and M2 macrophage polarization and also elicited cartilage regeneration (Chen et al., 2019a). Like cell-free therapies based on EVs, few EV-based tissue engineering strategies have been investigated in the field of cartilage regeneration, and the course to new discoveries remains.

All the presented preclinical studies show that EVs embody a great hope to become part of the next-generation treatments in regenerative medicine for articular cartilage regeneration.

## Author Contributions

ÉV and MC drafted the manuscript and gave the final approval of the version to be published. AB designed the figures. ÉV, HM, JV, AB, and MC helped to write, discuss, and edit the manuscript. All authors contributed to the article and approved the submitted version.

## Conflict of Interest

The authors declare that the research was conducted in the absence of any commercial or financial relationships that could be construed as a potential conflict of interest.

## Footnotes

1 https://clinicaltrials.gov
